# Cryptic Species Diversification of the *Pedicularis siphonantha* Complex (Orobanchaceae) in the Mountains of Southwest China Since the Pliocene

**DOI:** 10.3389/fpls.2022.811206

**Published:** 2022-03-24

**Authors:** Rong Liu, Hong Wang, Jun-Bo Yang, Richard T. Corlett, Christopher P. Randle, De-Zhu Li, Wen-Bin Yu

**Affiliations:** ^1^Center for Integrative Conservation, Xishuangbanna Tropical Botanical Garden, Chinese Academy of Sciences, Mengla, China; ^2^Center of Conservation Biology, Core Botanical Gardens, Chinese Academy of Sciences, Mengla, China; ^3^University of Chinese Academy of Sciences, Beijing, China; ^4^Key Laboratory for Plant Diversity and Biogeography of East Asia, Kunming Institute of Botany, Chinese Academy of Sciences, Kunming, China; ^5^Plant Germplasm and Genomics Centre, Germplasm Bank of Wild Species, Kunming Institute of Botany, Chinese Academy of Sciences, Kunming, China; ^6^Department of Biological Sciences, Sam Houston State University, Huntsville, TX, United States; ^7^Southeast Asia Biodiversity Research Institute, Chinese Academy of Sciences, Yezin, Myanmar

**Keywords:** *Pedicularis siphonantha* complex, phylogenetic delimitation, speciation, mountains of Southwest China, the Hengduan Mountains

## Abstract

Morphological approaches often fail to delimit species in recently derived species complexes. This can be exacerbated in historical collections which may have lost key features in specimen preparation and preservation. Here, we examine the *Pedicularis siphonantha* complex, endemic to the Mountains of Southwest China. This complex is characterized by its red/purple/pink and long-tubular corolla, and twisted, beaked galea. However, herbarium specimens are often difficult to identify to species. Molecular approaches using nrITS or nuclear ribosomal internal transcribed spacer (nrITS) + plastid DNA (ptDNA) have been successfully used for species identification in *Pedicularis*. To resolve taxonomic confusion in the *Pedicularis siphonantha* complex, we reconstructed phylogenies of the complex using nrITS and four plastid DNA loci (*matK*, *rbcL*, *trnH-psbA*, and *trnL-F*). To recover as much of the phylogenetic history as possible, we sampled individuals at the population level. Topological incongruence between the nrITS and ptDNA datasets was recovered in clades including two widely distributed species, *Pedicularis milliana* and *Pedicularis tenuituba*. Based on morphological, geographical, and genetic evidence, we suggest that hybridization/introgression has occurred between *P. milliana* and *Pedicularis sigmoidea*/*Pedicularis* sp. 1 in the Yulong Snow Mountain of Lijiang, northwest Yunnan, and between *P. tenuituba* and *Pedicularis leptosiphon* in Ninglang, northwest Yunnan. After removing conflicting DNA regions in *Pedicularis dolichosiphon* (nrITS) and *P. milliana* (ptDNA), the concatenated nrITS and ptDNA phylogenies distinguish 11 species in the *P. siphonantha* complex, including two undescribed species, from the Jiaozi and Yulong Snow Mountains, respectively. Phylogeographical analyses indicate that the *P. siponantha* complex originated from south of the Hengduan Mountains, expanding north to the Himalayas and the Yunnan-Guizhou Plateau. Moreover, the uplift of the Qinghai-Tibet Plateau and climate oscillations may have driven further diversification in the complex.

## Introduction

The Mountains of Southwest China host one of the richest temperate floras, with a high proportion of endemic species (Boufford, 2014). Mountain uplifts and the monsoon climate create geographically and ecologically isolated habitats, which have driven plant diversification in this region (Hoorn et al., 2013; Xing and Ree, 2017; Ding et al., 2020). Rapid diversification and frequent introgression compound taxonomic confusion, as documented in megadiverse genera of the region, such as *Meconopsis* Vig. (Papaveraceae), *Primula* L. (Primulaceae), and *Rhododendron* L. (Ericaceae) (Zha et al., 2010; Yang et al., 2012; Favre et al., 2016). Species delimitation is traditionally based on morphological characters. However, morphological approaches often fail to delimit recently diverged species, resulting in cryptic species complexes (Bickford et al., 2007; Struck et al., 2018). The study of cryptic species, therefore, offers a window into the diversification and the maintenance of recently divergent species groups.

**FIGURE 1:**
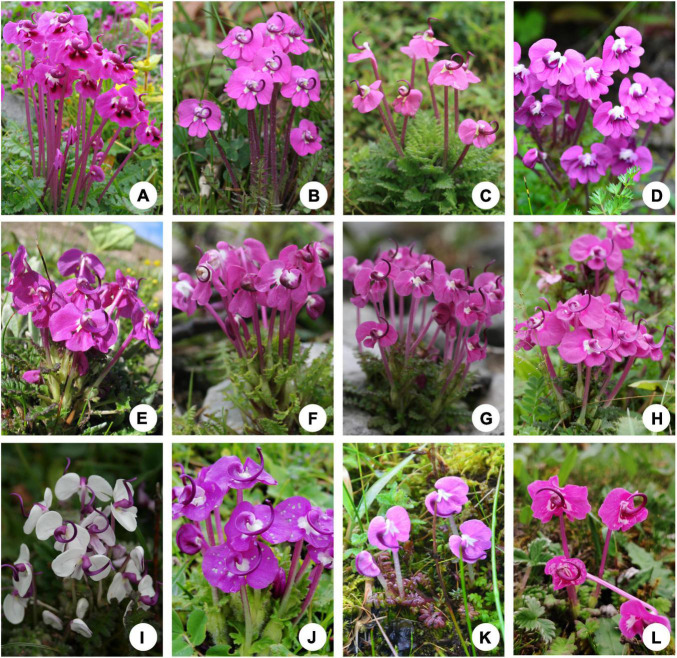
Field photos of 11 species of the *Pedicularis siphonantha* complex and *Pedicularis delavayi*, **(A)** *Pedicularis tenuituba* H. L. Li; **(B)** *Pedicularis leptosiphon* H. L. Li; **(C)** *Pedicularis dolichosiphon* (Hand.-Mazz.) H. L. Li; **(D)** *P. siphonantha* D. Don; **(E)** *Pedicularis* sp. 2 from Jiaozi snow mountain; **(F)** *Pedicularis milliana* W. B. Yu, D. Z. Li, and H. Wang; **(G)** *Pedicularis* sp. 1 from Ganheba, Lijiang; **(H)** *Pedicularis sigmoidea* Franch. ex Maxim; **(I)** *Pedicularis variegata* H. L. Li; **(J)** *Pedicularis dolichantha* Bonati; **(K)** *Pedicularis humilis* Bonati. **(L)** *Pedicularis delavayi* Franch. ex Maxim.

*Pedicularis* L. (Orobanchaceae) consists of approximately 600–800 species, of which two-thirds are endemic to the Mountains of Southwest China (Li, 1948, 1949). *Pedicularis* exhibits dramatic variations in corolla structure and galea form, beak length and shape, and corolla tube length, which are key characters for species delimitation. Four general corolla types are recognized: (A) short-tubular corolla with a beakless, toothless galea (upper lip), (B) short-tubular corolla with a toothed galea, (C) short-tubular corolla with a beaked galea, and (D) long-tubular corolla with a beaked galea (Maximowicz, 1888; Li, 1948, 1949; Tsoong, 1955, 1956, 1963). Long-tubular corollas always bear a beaked galea, which has been considered as a derived corolla type. Ree (2005) and Yu et al. (2015) have demonstrated that long-tubular corollas appear to have been derived from short-tubular corollas several times, resulting in taxonomic confusion. The *Pedicularis siphonantha* complex includes only long-tubular species with the purple-red corolla and an S-shaped, beaked galea (Figure 1). *Pedicularis siphonantha* D. Don from the Himalayas was the first described species. To date, at least 11 species of this complex are recognized from the western Himalayas to the Mountains of Southwest China (Li, 1949; Yang et al., 1998; Yu et al., 2015, 2018). The *P. siphonantha* complex was supported as monophyletic by Yu et al. (2015). However, species within this complex are difficult to distinguish, especially as herbarium specimens, which lose three-dimensional corolla structure and color and often lack field photos and descriptions of key diagnostic characters. This results in uncertainty about the number of species and their geographical distributions. For example, pressed specimens of *Pedicularis delavayi* Franch. ex Maxim. resemble *P. siphonantha*, though the three-dimensional structure of the middle lobe of the lower lip and corolla throat color allows easy distinction. Because of the loss of structural and color characteristics in preserved specimens, Tsoong (1963) inferred that the distribution of *P. siphonantha* extended to the Mountains of Southwest China, and included the species as a variety of *P. siphonantha*, i.e., *P. siphonantha* var. *delavayi* (Franch. ex Maxim.) P. C. Tsoong. In contrast, Li (1949) considered *P. delavayi* as a separated species. Li (1949) and Tsoong (1963), as well as Yang et al. (1998) and other botanists, misidentified most herbarium specimens of *Pedicularis tenuituba* H. L. Li and *Pedicularis milliana* W. B. Yu et al. as *P. delavayi* (Yu et al., 2018). In addition, the infraspecific taxonomy of *P. siphonantha* is not fully resolved yet in the Himalaya region (Yu et al., 2018).

**FIGURE 2:**
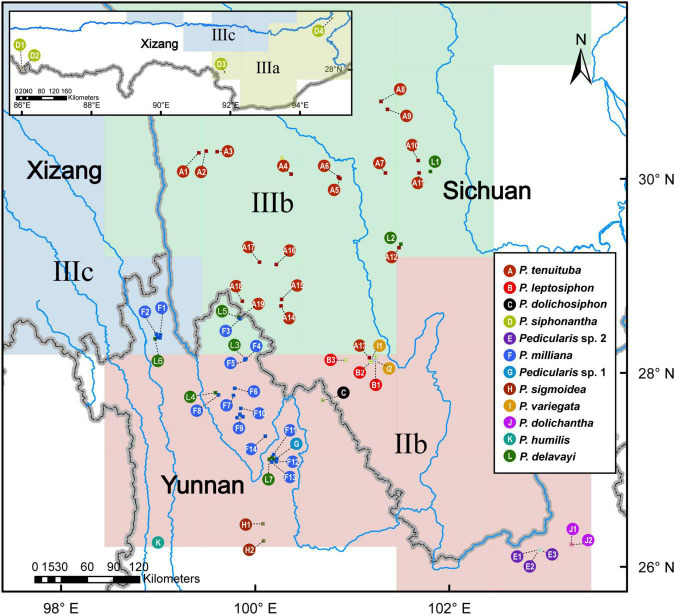
A geographical sampling of the *Pedicularis siphonantha* complex from the Hengduan Mountains, southwestern China. The colors of circles correspond to different taxa. Numbers and letters indicate populations; these abbreviations are also used in Figures 3, 4, 6. More information regarding collection vouchers can be found in Supplementary Table 1.

Molecular approaches have been widely applied for species identification (Hebert et al., 2003; Hebert and Gregory, 2005; Hollingsworth et al., 2016; Kress, 2017). Four candidate DNA barcodes [nuclear ribosomal internal transcribed spacer (nrITS), and three plastid *matK*, *rbcL*, and *trnH-psbA* regions] can be used to discriminate more than 89.0% of *Pedicularis* species (Yu et al., 2011; Liu et al., 2013). Based on nrITS and four plastid loci (*matK*, *rbcL*, *trnH-psbA*, and *trnL-F*), no samples of *P. delavayi* cluster with other species of the *P. siphonanta* complex. Yu et al. (2018) have reinstated *P. delavayi* as a separate species and discovered an undescribed species, *P. milliana*, which was previously misidentified as *P. siphonantha* var. *delavayi* or *P. delavayi*. To date, species delimitation of the *P. siphonantha* complex is not fully resolved, due to limited sampling and poorly known species distributions (Li, 1949; Yu et al., 2015, 2018). For example, though *P. milliana* occurs widely in northwest Yunnan and *Pedicularis sigmoidea* Franch. ex Maxim. occurs in the south margin of *P. milliana* (Figure 2), specimens (see Figure 1G) from the Yulong Snow Mountain (G1 in Figure 2) bear a remarkable S-shaped beak similar to that of *P. sigmoidea*. Other specimens (see Figure 1E), from the Jiaozi Snow Mountain (E1-3 in Figure 2), appear to be distinct from known species (Yu et al., 2015, 2018). Morphological ambiguity is supported by topological incongruence between nrITS and plastid gene datasets (Yu et al., 2015), which might be caused by hybridization or introgression.

**FIGURE 3:**
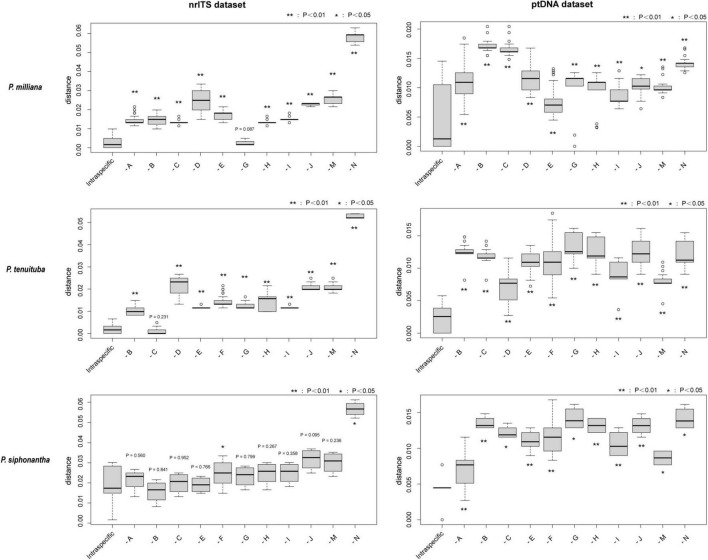
K-S test for intraspecific and interspecific genetic distance. The intraspecific genetic distance of *Pedicularis milliana*, *Pedicularis tenuituba*, and *Pedicularis siphonantha* are estimated. Statistical significance was shown on every group (**P < 0.01, *P < 0.05). -A: to *Pedicularis tenuituba*; -B: to *Pedicularis leptosiphon*; -C: to *Pedicularis dolichosiphon*; -D: *to Pedicularis siphonantha*; -E: to *Pedicularis* sp. 2 from Jiaozi Snow Mountain; -F: to *Pedicularis milliana*; -G: to *Pedicularis* sp. 1 from Ganheba; -H: to *Pedicularis sigmoidea*; -I: to *Pedicularis variegata*; -J: to *Pedicularis dolichantha*; -M: to *Pedicularis amplituba* (outgroup); -N: to *Pedicularis tahaiensis* (outgroup).

**FIGURE 4:**
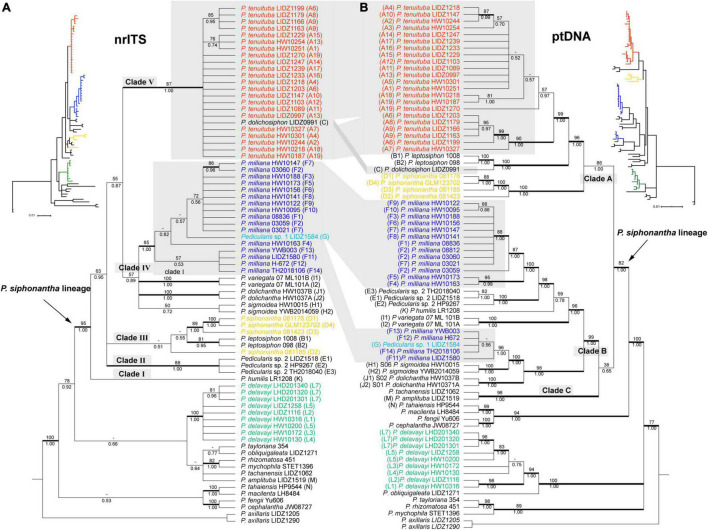
Bayesian Inference (BI) tree comparisons of *Pedicularis siphonantha* complex inferred from nrITS **(A)** and the concatenated chloroplast **(B)** datasets. Phylogenetic incongruence is marked by shadow, Numbers associated with the branches are bootstrap value (BS) and BI posterior probabilities (PP), and thicker lines are indicated as BS ≥ 70 and PP ≥ 0.95. Node with BS < 50 was collapsed.

**FIGURE 5:**
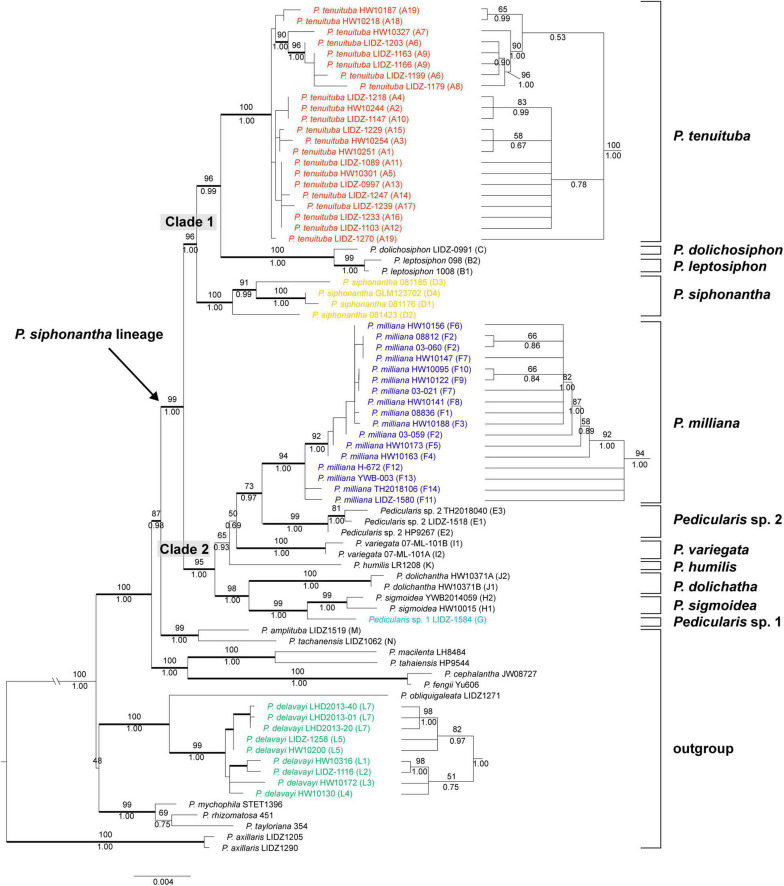
Bayesian inference (BI) tree of the *Pedicularis siphonantha* complex inferred from the modified nrITS+ptDNA dataset by removing the conflicting sequence nrITS of *Pedicularis dolichosiphon* (nrITS) and the four ptDNA regions (*matK*, *rbcL*, *trnH-psbA*, and *trnL-F*) of the samples F11-F14 of *Pedicularis milliana* in accordance with the topological incongruence between the nrITS and ptDNA phylogenies (see Figure 4 and Table 3). Numbers associated with the branches are ML BS value and BI PP, and thicker lines indicate BS ≥ 70 and PP ≥ 0.95. Node with BS < 50 was collapsed. The topology of the *P. milliana* clade with short branch lengths appear on the right.

**TABLE 1:** The best-fit model of partition dataset partitions.

DNA marker	nrITS	ptDNA			
		*matK*	*rbcL*	*trnH-psbA*	*trnL-F*
BIC model	GTR+G4	HKY+G4	K80+I	F81+G4	GTR+I+G4
-lnL	2248.9747	1785.4927	1244.1168	1854.9583	2345.2173
K	9	5	2	4	10
Frequency A	0.1973	0.3577	0.2500	0.3973	0.3678
Frequency C	0.2946	01791	0.2500	0.1061	0.1663
Frequency G	0.2802	0.1725	0.2500	0.1037	0.1527
A↔C	0.9500	1.0000	1.0000	1.0000	1.7725
A↔G	0.9996	3.5998	4.0355	1.0000	1.4874
A↔T	1.3845	1.0000	1.0000	1.0000	0.2393
C↔G	0.2899	1.0000	1.0000	1.0000	0.5144
C↔T	3.5963	3.5998	4.0355	1.0000	1.5535
G↔T	1.0000	1.0000	1.0000	1.0000	1.0000
Gamma distribution shape parameter of variable sites	0.2744	0.2797	0.0000	0.2844	0.5634
Proportion of invariable sites	0.0000	0.0000	0.8783	0.0000	0.6024

**TABLE 2:** Sequence characteristics of nrITS and four plastid DNA regions.

Parameters	*n*	nrITS	Plastid DNA loci				Concatenated datasets	Total
			*matK*	*rbcL*	*trnH-psbA*	*trnL-F*		
No. of accessions		77	77	77	71	76	78	78
Aligned length(bp)		723	856	727	725	987	3295	4,018
**Variable sites/Parsimony informative sites**								
*P. siphonantha* complex + Outgroups	78	150/103	127/70	43/31	128/99	135/89	417/388	566/490
*P. delavayi*	9	5/1	1/0	3/2	9/3	13/3	37/7	42/8
*P. siphonantha* complex	57	61/39	84/40	33/21	86/72	73/47	259/179	319/217
*P. tenuituba*	22	7/3	13/5	9/2	9/4	10/7	40/16	59/19
*P. milliana*	17	8/4	14/10	10/6	22/22	15/13	64/52	70/55
*P. leptosiphon*	2	1/0	0/0	3/0	0/0	1/0	4/0	5/0
*P. siphonantha*	4	19/7	10/1	0/0	12/0	11/1	33/2	52/9
*Pedicularis* sp. 2	3	1/0	4/0	0/0	1/0	4/0	7/0	7/0
*P. variegata*	2	0/0	0/0	0/0	0/0	4/0	4/0	4/0
*P. sigmoidea*	2	6/0	3/0	0/0		0/0	7/0	13/0
*P. dolichantha*	2	0/0	0/0	0/0	0/0	3/0	3/0	3/0

**TABLE 3:** Summary of the Shimodaira–Hasegawa (SH) and the Approximately Unbiased (AU) tests.

Topological constraint	LnL	deltaL	p-AU	p-SH
ptDNA dataset	−7319.538			
Best tree of the nrITS phylogeny	−8004.596	685.060	<0.001	<0.001
Constraint the monophyly of *P. milliana*	−7412.726	93.188	<0.001	0.005
Constraint the clade *P. tenuituba + P. dolichosiphon*	−7319.538	0.000	0.545	1.000
Constraint the monophyly of the *P. siphonantha* complex	−7319.539	0.001	0.455	0.451
nrITS dataset	−2255.921			
Best tree of the ptDNA phylogeny	−2403.898	147.980	<0.001	<0.001
Constraint the monophyly of *P. tenuituba*	−2275.090	19.320	0.005	0.001
Constraint *P. tenuituba* + *P. leptosiphon* + *P. siphonantha*	−2255.921	0.000	0.988	1.000
Constraint *Pedicularis* sp. 2 (Jiaozi Mountain) sister to *P. milliana*	−2266.434	10.513	0.0147	0.569
Constraint *P. tachanensis* + *P. amplituba* sister to clade IV	−2282.702	26.782	0.002	0.248
Concanated nrITS + ptDNA dataset	−10765.132			
Constraint *P. tachanensis* + *P. amplituba* sister to clade 2	−10782.516	17.384	0.005	0.049

*deltaL, logL difference from the maximal logL in the set.*

**FIGURE 6:**
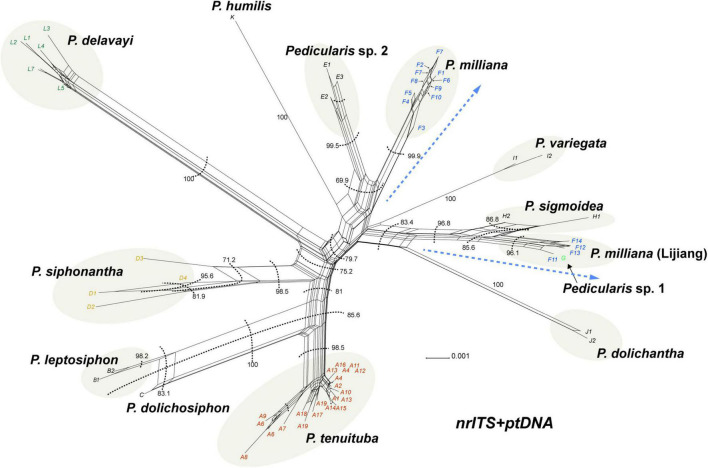
Neighbor-net analysis of *Pedicularis siphonantha* complex using complete nrITS + ptDNA datasets. Bootstrap support values for clusters are indicated next to the respective cluster delimitation (dashed lines); blue dashed lines indicate the split of *Pedicularis milliana.*

In this study, we reconstructed a comprehensive phylogeny of the *P. siphonantha* complex using five DNA loci (nrITS, *matK, rbcL, trnH-psbA*, and *trnL-F*) with population-level sampling. Our main goals were to: (1) explore patterns and causes of phylogenetic incongruence between nrITS and plastid DNA datasets in the *P. siphonantha* complex; (2) revise species delimitations in the *P. siphonantha* complex; and (3) investigate the causes of species diversification in the *P. siphonantha* complex.

## Materials and Methods

### Taxon Sampling

We sampled 78 individuals, mainly from the Hengduan Mountains, as well as the Himalayas and the Yunnan-Guizhou Plateau, representing 11 taxa of the *P. siphonantha* complex and 12 other *Pedicularis* species (Supplementary Table 1). The 11 taxa covered nine recognized species of the complex, with the exception of *Pedicularis fastigiata* Franch., which is known only from the type collection by Orléans H.d’ s.n. (P, barcode P00520823) of the 78 individuals, 50 were newly sampled and sequenced. There were 22 samples (19 populations) of *P. tenuituba*, widely distributed in western Sichuan, and 17 samples (14 populations) of *P. milliana*, endemic to the northwestern Yunnan. Three populations of *P. milliana* (i.e., F11, F12, and F13) were collected from the Yulong Snow Mountain in Lijiang, and population F14 was collected from the Haba Snow Mountain in Shangri-La. In addition, population G (i.e., sample LIDZ1584), collected from Ganheba in the Yulong Snow Mountain represents an unknown taxon, which is similar to *P. sigmoidea* in the shape of the galea beak but has a smaller corolla. Populations E1–E3, collected from the Jiaozi Snow Mountain, represents another unknown taxon, which is distinguished from *P. milliana* by its oblate and crested beak. The remaining six taxa of the *P. siphonantha* complex have narrow distributions, so only a few individuals/populations were included in this study. We included nine samples (seven populations) of *P. delavayi* from the northwestern Yunnan and western and northern Sichuan, where it overlaps with *P. milliana* and *P. tenuituba*. Geographic information for all samples the *P. siphonantha* complex is shown in Figure 2.

### Molecular Methods

The nrITS and four plastid DNA (ptDNA) markers (*matK*, *rbcL*, *trnH-psbA*, and *trnL-F*) were amplified and sequenced in this study. Primer information of five DNA markers were presented in previous studies (Yu et al., 2011, 2013, 2018). Genomic DNA of 50 new samples was extracted using a modified CTAB method from silica gel–dried leaves. PCR amplification and sequencing profile followed Yu et al. (2011). Raw sequences were assembled and edited using Geneious 7.1 (Kearse et al., 2012). The nrITS region has multiple copies in the genome. These copies showed evolutionary consistent in the newly sequenced 46 samples, only one sample (*P. milliana* F2/03-060) had two ambiguous basecalls (i.e., multiple superimposed peaks in chromatograms), and three samples (*P. milliana* F2/03-059, *P. tenuituba* A11/HW10187, and *P. tenuituba* A7/HW10327) had one basecall. The ambiguous site was assigned using IUPAC ambiguity characters. Assembled sequences were aligned using MAFFT 7.4 (Katoh et al., 2019), then adjusted manually using Geneious. Sequence characteristics and Kimura 2-parameter (K-2P) model-based genetic distances among taxa were calculated using MEGA 10.0 (Kumar et al., 2018), and non-parametric two-sample Kolmogorov–Smirnov (K–S) tests of genetic distances between and within species were estimated for widely distributed species *P. tenuituba*, *P. milliana*, and *P. siphonantha* using SPSS 25.0 (IBM, 2017). The nrITS and ptDNA datasets were analyzed separately.

### Phylogenetic Analyses

Both Bayesian Inference (BI) and maximum likelihood (ML) were used to reconstruct phylogenetic relationships in the *P. siphonantha* complex. To explore topological incongruence between nrITS and the concatenated ptDNA phylogenies, the two datasets were analyzed separately. Before concatenating the data sets, we removed sequences that seemed to be the source of conflict. The concatenated datasets (ptDNA and nrITS + ptDNA) were partitioned by gene and the best-fit model was estimated using Modeltest-ng (Darriba et al., 2020) (see Table 1). BI Markov chain Monte Carlo (MCMC) analyses were performed using MrBayes 3.2 (Ronquist et al., 2012) for 2,000,000 generations with two simultaneous runs, each comprising four incrementally heated chains. BI analyses were started with random trees and sampled every 1,000 generations. The first 25% of trees were discarded as burn-in, and the remaining trees were used to generate a majority-rule consensus tree. Posterior probability (PP) values ≥0.95 were considered as well-supported (Alfaro et al., 2003; Kolaczkowski and Thornton, 2007). ML tree search was performed using RAxML version 8.2.12 (Stamatakis, 2014) under GTR + Γ. Node support was evaluated using 1,000 non-parametric bootstrap (BS) replicates. Nodes with BS values ≥70 were considered well-supported (Hillis and Bull, 1993).

To explore patterns of introgression in the complex, we constructed phylogenetic networks of the *P. siphonantha* complex based on the total dataset by the concatenation of all nrITS and ptDNA sequences by using SplitsTree 4.14.1 (Huson and Bryant, 2006). The Neighbor-net model was performed using the Kimura 2-parameter (K-2P) distance and Ordinary Least Squares Method, with 1,000 BS replicates to estimate split support. Splits with BS ≥ 70 were considered as well-supported.

### Topological Conflict Analyses

Thresholds of PP ≥ 0.95 and BS ≥ 70 were interpreted as identifying incongruent clades between the nrITS and ptDNA datasets. Based on topological incongruence between the nrITS and ptDNA datasets, the DNA sequence would be considered as heterogeneous one if the phylogenetic cluster was not consistent with the morphological cluster, then the heterogeneous sequence was removed from the concatenated nrITS + ptDNA dataset. Herein, the nrITS sequence of *Pedicularis dolichosiphon* (Hand.-Mazz.) H. L. Li and the four ptDNA regions (*matK*, *rbcL*, *trnH-psbA*, and *trnL-F*) of the samples F11–F14 of *P. milliana* were identified as heterogeneous sequences, so that those sequences were removed from the concatenated nrITS + ptDNA dataset. Then, the concatenated nrITS + ptDNA phylogeny was performed using the same methods as nrITS and the concatenated ptDNA phylogenetic analyses (see above). Additionally, the Shimodaira–Hasegawa (SH) test (Shimodaira and Hasegawa, 1999) and the approximately unbiased (AU) test (Shimodaira, 2002) were used to estimate the degree of topological incongruence among the three datasets (nrITS, ptDNA, and modified nrITS + ptDNA). Constraint trees were constructed in Mesquite version 3.6 (Maddison and Maddison, 2019), and the SH and AU tests were performed using IQ-Tree 1.6 (Lam-Tung et al., 2015).

### Phylogeographical Analyses

Ancestral geographical distributions were inferred using BEAST 2.6.3 (Bouckaert et al., 2014). The concatenated nrITS and ptDNA dataset, including all samples of the *P. siphonantha* complex and two outgroups *Pedicularis amplituba* H. L. Li and *Pedicularis tachanensis* Bonati, was imported into BEAUti with “beast-classic package” (Lemey et al., 2009). Samples were assigned to one of four subregions of the Sino-Himalayan flora following Liu et al. (2021). Taking into account the center of endemism identified by Zhang et al. (2016), two samples of *Pedicularis siphonantha* (D1, D2) collected from Yadong country in the middle Himalaya were assigned to subregion IIIa. The sites model was calibrated using the “bModeltest package” (Bouckaert and Drummond, 2017), the molecular clock model was set to “Relaxed Clock Log Normal,” and the Yule model served as the tree prior. The divergence time of the most recent ancestor between the *P. siphonantha* complex and the two outgroups was constrained to 9.1 ± 2 Mya. A second calibration point was obtained from the analysis of Yu et al. (2015) (Supplementary Figure 1). MCMC chains were run for 10,000,000 generations, with parameter values and trees sampled every 1,000 generations. Effective sample size (ESS > 200) was assessed using Tracer 1.7 (Rambaut et al., 2018). After discarding 25% of the initial trees as burn-in, the maximum clade credibility (MCC) tree with mean ages and 95% highest posterior density (HPD) intervals on nodes was reconstructed using TreeAnnotator 2.6.3 (Bouckaert et al., 2014).

## Results

### Matrix Characteristics

Matrix characteristics of nrITS, four plastid DNA, and the concatenated ptDNA datasets are shown in Table 2. Similar to previous studies (Yu et al., 2011, 2013, 2015, 2018), these five loci included an adequate numbers of variable sites and parsimony-informative sites for subsequent phylogenetic analyses in the *P. siphonantha* complex. The nrITS dataset is the most informative, followed by two plastid intergenic spacer datasets (*trnH-psbA* and *trnL-F*). The two protein-coding genes (*rbcL* and *matK*) are less informative than the three spacer datasets. Sequences of *P. siphonantha* and *P. milliana* show the highest variation at the species level for both nrITS and ptDNA datasets.

### Genetic Distance Estimation

Comparisons of genetic distances within and between species using the K–S test are shown in Figure 3. The intraspecific genetic distances among *P. milliana* sequences were significantly smaller (*P <* 0.05) than the distance between *P. milliana* and any other taxon, with one exception; nrITS sequences of *P. milliana* and *Pedicularis* sp. 1 did not differ significantly. The same pattern holds for *P. tenuituba*, with the exception being a non-significant distance with nrITS sequences *P. dolichosiphon*. While the ptDNA distances within *P. siphonantha* were significantly less than the distance between *P. siphonantha* and ptDNA of any other taxon, distances of nrITS sequences within *P. siphonantha* were significantly smaller than the distance between *P. milliana* and *Pedicularis tahaiensis* Bonati (*P* < 0.05).

### Phylogenetic Analyses of the nrITS Dataset

Maximum likelihood and BI analyses obtained identical topologies for the nrITS dataset (Figure 4A). The *P. siphonantha* complex was recovered as monophyletic (BS/PP = 95/1.00), and *P. delavayi* was not included in that clade. Within the *P. siphonantha* complex, five major clades were recovered. Clade I had only *Pedicularis humilis* Bonati, and it was moderately supported as sister to the remaining four clades (BS/PP = 63/0.95). The relationships among Clades II–V were not well-resolved. Clade II consisted of three individuals of *Pedicularis* sp. 2, and clade III included two species, *Pedicularis leptosiphon* H. L. Li and *P. siphonantha*, with *P. leptosiphon* nested within *P. siphonantha*, although with weak support. Clades IV and V were weakly supported as sister lineages (BS/PP = 55/0.87). Clade IV consisted of five species, *Pedicularis dolichantha* Bonati, *P. milliana*, *P. sigmoidea, Pedicularis variegata* H. L. Li, and *Pedicularis* sp. 1 (G). In this clade, *Pedicularis* sp. 1 (G) was nested within *P. milliana*. Clade V consisted of *P. dolichosiphon* nested within 22 samples of *P. tenuituba*.

### Phylogenetic Analyses of the ptDNA Dataset

Maximum likelihood and BI obtained identical topologies from the ptDNA dataset (Figure 4B). Unlike the nrITS phylogenies, the *P. siphonantha* complex was not recovered as monophyletic, including *P. amplituba* + *P. tachanensis* not considered here to be members of the complex. The core species of the *P. siphonantha* complex were split into two major clades (i.e., Clades A and B + C), with Clade C (*P. amplituba* and *P. tachanensis*) weakly supported as sister to the remaining members of Clade B (BS/PP = 38/0.65). Clade A included four species, *P. dolichosiphon*, *P. leptosiphon*, *P. siphonantha*, and *P. tenuituba*, which were strongly supported as monophyletic (BS/PP ≥ 99/1.00). Within Clade A, *P. siphonantha* was sister to the remaining three species with *P. dolichosiphon* and *P. leptosiphon* forming a clade sister to *P. tenuituba*.

Clade B contained seven species, which were split into two subclades. One subclade included *P. dolichantha*, *P. sigmoidea*, *Pedicularis* sp. 1, and four samples of *P. milliana* from the Yunlong Snow Mountain and the Haba Snow Mountain. In this subclade, *P. dolichantha* was sister to the remaining species, with *P. sigmoidea* as the sister to a clade including *Pedicularis* sp. 1 nested within *P. milliana*. The other subclade included *P. humilis*, *P. variegata*, *Pedicularis* sp. 2, and 13 samples of *P. milliana*, which were all supported as monophyletic. Within this subclade, *P. variegata* was sister to the remaining species, with *P. humulis* arising as sister to a clade including *Pedicularis* sp. 2 + *P. milliana*.

### Topological Conflicts Between the nrITS and ptDNA Phylogenies

NrITS and ptDNA phylogenies are incongruent in the placement of *P. milliana*, *P. tenuituba*, and their relatives. In the nrITS phylogeny, 17 samples of *P. milliana* were only monophyletic if they included *Pedicularis* sp. 1 (BS/BP = 85/1.00). In the ptDNA phylogeny, they were separated into two distant clades, i.e., four samples (F11–F14) formed a clade by including *Pedicularis* sp. 1 (BS/BP = 100/1.00) as sister to *P. sigmoidea*, and the remaining 13 samples were monophyletic (BS/BP = 96/1.00) as sister to *Pedicularis* sp. 2. Twenty-four samples of *P. tenuituba* are strongly supported as monophyletic (BS/BP = 99/1.00) in the ptDNA phylogeny, but they are made paraphyletic by the inclusion of *P. dolichosiphon* in the nrITS phylogeny. Therefore, the four ptDNA regions of *P. milliana* samples F11–F14 and the nrITS sequence of *P. dolichosiphon* were identified as heterogeneous sequences. In addition, the nrITS dataset supports monophyly of the *P. siphonantha* complex (BS/BP = 95/100), but the ptDNA dataset does not, by including two short-tubular species *P. amplituba* and *P. tachanensis*.

Results of the AU and SH test for alternative hypotheses are summarized in Table 3. If phylogeny is constrained by the ptDNA dataset, SH and AU tests rejected (*P* < 0.01) the best tree topology of the nrITS dataset and monophyly of *P. milliana*. These tests failed to reject the monophyly of *P. tenuituba* + *P. dolichosiphon* and monophyly of the *P. siphonantha* complex. Meanwhile, when constrained by the nrITS dataset, SH and AU tests failed to reject (*P* > 0.05) the monophyly of *P. tenuituba* + *P. leptosiphon* + *P. siphonantha*, and the monophyly of *P. milliana* + *Pedicularis* sp. 2, but rejected the best tree topology of the ptDNA dataset, and the short tubular *P. tachanensis* + *P. amplituba* clade sister to the Clade B. Moreover, for the modified nrITS + ptDNA dataset, the null hypothesis that short tubular *P. tachanensis* + *P. amplituba* is sister to Clade 2 was rejected (*P* < 0.05).

### Phylogenetic Analyses of the Concatenated nrITS + ptDNA Dataset

After removing conflicting sequences, *P. dolichosiphon* (nrITS) and *P. milliana* (ptDNA regions of F11–F14), ML and BI analyses produced nearly the same topology (Figure 5). Within the strongly monophyletic (BS/BP = 99/1.00) *P. siphonantha* complex, there were two major clades. Clade 1 (BS/BP = 96/1.00) included *P. dolichosiphon*, *P. leptosiphon*, *P. siphonantha*, and *P. tenuituba*. In this clade, *P. siphonantha* was sister to the remaining taxa, with the monophyletic *P. tenuituba* (BS/BP = 100/1.00) sister to a clade including *P. dolichosiphon* + *P. leptosiphon* (BS/BP = 100/1.00). Clade 2 (BS/BP = 95/1.00) included six taxa forming two subclades. In the larger subclade, *P. milliana* was monophyletic (BS/BP = 94/1.00), and sister to *Pedicularis* sp. 2 (BS/BP = 99/1.00). A clade including *P. milliana* + *Pedicularis* sp. 2 + *P. variegata* was sister to *P. humilis* (BS/BP = 65/0.93). In the other subclade, *P. dolichantha* was sister to *P. sigmoidea* + *Pedicularis* sp. 1 (BS/BP = 99/1.00).

### Phylogenetic Network of the *Pedicularis siphonantha* Complex

The phylogenetic network of the concatenated nrITS and ptDNA dataset showed *P. milliana* split into two clusters (Figure 6), identical to those recovered in the ptDNA topology. Four samples from the Yulong Snow Mountain and the Haba Snow Mountain were nested with *Pedicularis* sp. 1 as sister to *P. sigmoidea* (BS = 96.8), and the other 13 samples were monophyletic as sister to *Pedicularis* sp. 2. In addition, *P. dolichosiphon* was resolved as either sister to *P. leptosiphon* (BS = 100) or *P. tenuituba* (BS = 85.6).

### Phylogeographical Analyses of the *Pedicularis siphonantha* Complex

**FIGURE 7:**
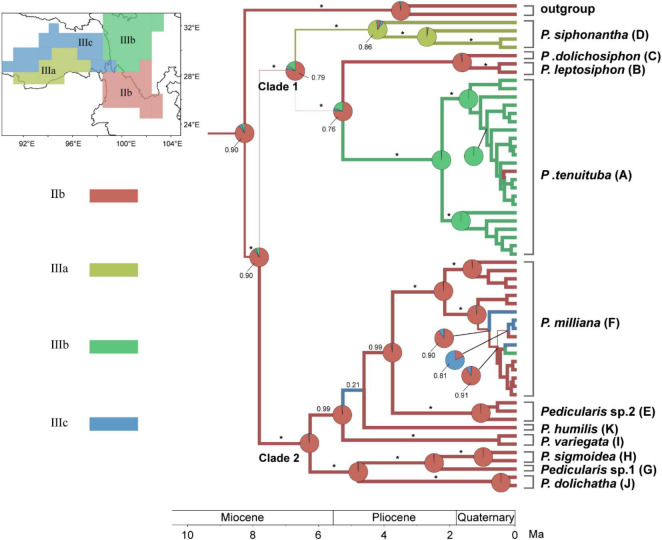
Bayesian phylogeographical reconstruction of ancestral area of the *Pedicularis siphonantha* complex in the Sino-Himalayan region using the modified nrITS + ptDNA dataset (see above). Pie charts and thickness of branch represent the marginal probabilities for potential ancestral areas with each subregion (Liu et al., 2021), represented by a different color. BI PPs are above branches (* = 1.00).

Phylogeographical analysis indicated that the most common ancestor of the *P. siphonantha* complex diverged from other *Pedicularis* in the late Miocene (6.04Mya–10.38Mya), south of the Hengduan Mountains (IIb), which harbors nine of eleven species/taxa of this complex (Figure 7). Species diversification of this complex mainly occurred in the Pliocene (2.48Mya–5.3Mya). After the initial divergence of Clades 1 and 2, members of Clade 1 migrated to the north and west, with Clade 2 diversifying *in situ*. In the Clade 1, *P. siphonantha* diverged and diversified in the Himalayan region (IIIa), *P. leptosiphon* and *P. dolichosiphon* diverged *in situ* (IIb), and *P. tenuituba* diverged north of the Hengduan Mountains. In Clade 2, seven species/taxa diverged *in situ* (IIb), with some populations of *P. milliana* migrating northward to IIIb and IIIc.

## Discussion

### Topological Incongruence Between the nrITS and ptDNA Phylogenies

Topological incongruence between nuclear/nrITS and ptDNA phylogenies has been reported in many taxa (Rieseberg et al., 1996; Buckley et al., 2006; Stegemann et al., 2012; Yi et al., 2015; Stull et al., 2020; Ye et al., 2021). In this study, incongruences between the nrITS and ptDNA datasets were found among species within the *P. siphonantha* complex and in the sister relationship between the *P. siphonantha* complex and *P. amplituba* + *P. tachanensis* in phylogenetic analyses, as well as the estimation of genetic distance. The incongruence could be caused by convergent sequence evolution, incomplete lineage sorting, hybridization/introgression, horizontal gene transfer, and gene duplication/loss (Rokas et al., 2003; Degnan and Rosenberg, 2009). Phylogenetic network analyses suggested that introgression between *P. milliana* and *P. sigmoidea* and between *P. leptosiphon* and *P. tunuituba* is the most plausible explanation for discordance. However, incomplete lineage sorting, convergent sequence evolution, or others cannot be ruled out (Joly et al., 2009).

Introgression might have been common within recently derived species complexes when their distributions overlap (Acosta and Premoli, 2010; Liu et al., 2017; Liu et al., 2020). *Pedicularis* spp. are outcrossed and exclusively pollinated by bumblebees, and pollinator-mediated interspecific gene flow may cause hybridization and introgression among *Pedicularis* species in the same community (Hong and Li, 2005; Yang et al., 2007; Eaton et al., 2012). In the case of *Pedicularis* sect. *Cyathophora*, Yu et al. (2013) have documented that the plastid genome of *P. cyathophylloides* was likely captured from an ancestor of *P. cyathophylla* in the West Sichuan. Similarly, our results show that paraphyletic *P. milliana* populations were associated with distinct geographical ranges, suggesting that either genetic divergence occurred between two clusters due to allopatry and/or a plastid genome capture event. We, therefore, propose an ancient hybridization event between the ancestors of *P. milliana* (♀) and *P. sigmoidea/Pedicularis* sp. 1 (♂) in the Yulong Snow Mountain. In this scenario, high-altitude (≥3,800 m) hybrids (♀) backcrossed with ancestors of *P. milliana* (♀), and low-altitude (<3,800 m) hybrids became established as species at lower altitudes. Therefore, morphological consistency was found among high-altitude populations of *P. milliana*, while low altitude *Pedicularis* sp. 1 diverged from *P. milliana* in the shape of the beak and low lip of corolla. Sample C of *P. dolichosiphon* might also be the result of introgression between *P. tenuituba* (♀) and *P. leptosiphon* (♂), but greater population-level sampling is required to investigate this fully. In addition, more genomic evidence from organelle and nuclear genomes were needed to test these speculations.

### Phylogenetic Species Delimitation in the *Pedicularis siphonantha* Complex

Traditionally, the *P. siphonantha* complex together with other long-tubular species belonged to Ser. *Longiflorae* (Li, 1949; Tsoong, 1956). Phylogenetic analyses showed that Ser. *Longiflorae* is polyphyletic, but the *P. siphonantha* complex was monophyletic (Yu et al., 2015, 2018). In this study, ptDNA phylogenies rejected the monophyly of the *P. siphonantha* complex by including two short-tubular species, *P. amplituba* and *P. tachanensis*. The AU and SH tests also could not reject the inclusion of *P. tachanensis* + *P. amplituba* in the *P. siphonantha* complex using the nrITS dataset or the monophyly of the *P. siphonantha* complex using the ptDNA dataset. Moreover, phylogenies of the concatenated nrITS + ptDNA dataset strongly supported the monophyly of the *P. siphonantha* complex (BS/BP = 99/1.00). Therefore, the monophyly of the *P. siphonantha* complex should be accepted, but more nuclear genes and more robust phylogeny should be applied for evaluating this complex in the future.

Although introgression may confound phylogenetic species delimitation of *P. dolichosiphon* and *Pedicularis* sp. 1, species delimitations of the remaining nine species are well-resolved, which is consistent with morphological identification. For *Pedicularis* sp. 2, the corolla beak shape and lower-lip lobes are quite different than its sister species, *P. milliana*. Moreover, *Pedicularis* sp. 2 occurs on the Jiaozi Snow Mountain. Morphological, molecular, and biogeographic evidence all support *Pedicularis* sp. 2 to be a new species. It is worth noting that relatively high intraspecies genetic and phenotypic variations suggest *P. siphonantha* in the Himalayas needs further investigations [also reviewed by Yu et al. (2018)]. The phylogenetic position of *P. humilis* is still not well-resolved; however, it is an isolated species of the Gaoligong Mountain, perhaps the result of allopatric speciation.

Traditionally, morphological character similarity was the main evidence for assessing species relationships, but this criterion might be not suitable in the *P. siphonantha* complex. Because floral characters are labile in *Pedicularis*, morphologically similar species might be only distantly related. For example, phylogenetic analyses showed that *P. milliana* was clustered with morphologically different species including *P. variegata*, *P. humilis*, *P. dolichantha*, and *P. sigmoidea*, rather than the morphologically similar species *P. siphonantha*, contra previous placements [e.g., Li (1949); Tsoong (1963), and Yang et al. (1998)]. Moreover, the long-tubular species *P. delavayi* (excluded from the *P. siphonantha* complex) was clustered with short-tubular species *Pedicularis obiquigaleata* W. B. Yu and H. Wang. Understanding taxonomic affinities within the *P. siphonantha* complex requires morphological, geographical, and molecular evidence.

### Allopatric Speciation in the *Pedicularis siphonantha* Complex

Species diversification of *Pedicularis* in the Mountains of Southwest China is thought to be associated with the uplift of the Qinghai-Tibet Plateau and the establishment of the Asian monsoon climatic cycle (Ding et al., 2020). This rapid diversification resulted in dramatic variation in the form and shape of the corolla (Eaton et al., 2012), though the reasons for several independent transitions of the corolla tube from short to long remain unknown (Huang and Fenster, 2007; Yu et al., 2015, 2018; Huang et al., 2016). Macior and his colleagues (Macior, 1990; Macior and Tang, 1997; Macior et al., 2001) proposed that long-tubular corollas might have some adaptative advantages in the alpine meadow by extending the reproductive organs to attract bumblebee pollinators. The *P. siphonantha* complex, having long-tubular corollas, could be a good model for investigating species diversification in the Mountains of Southwest China. Phylogeographical analysis suggested that ancestors of the *P. siphonantha* complex originated from south of the Hengduan Mountains in the late Miocene, then rapidly expanded westward to the Himalayas, northward of the Hengduan Mountains, and eastward to the Yunnan-Guizhou plateau. Intense orogeny of southern Hengduan Mountains during the late Miocene and Pliocene (Lai et al., 2007; Wang et al., 2012; Favre et al., 2015; Zhang et al., 2017; Farnsworth et al., 2019) likely contributed to environmental heterogeneity driving rapid species divergence in the *P. siphonantha* complex. Therefore, *P. sigmoidea*, *P. dolichantha*, and *Pedicularis* sp. 2 are restricted to the margin of the Hengduan Mountains, while *P. milliana* and *P. tenuituba* in the heartland of the Hengduan Mountains, as well as *P. siphonantha* in the Himalayas, are widely distributed in the vast contiguous alpine meadows of those ranges.

Phylogeny of the modified nrITS + ptDNA dataset resolved the *P. siphonantha* complex as two major clades. In Clade 1, *P. siphonantha* is distributed in the western Himalaya (the type specimen was collected from Nepal), rather than widely distributed in the eastern Himalaya, Sichuan, and Yunnan, as described in the Flora of China (Yang et al., 1998); *P. leptosiphon* and *P. dolichosiphon* are restricted to Ninglang, Yunnan, and Muli, Sichuan; and *P. tenuituba* is widely distributed in western Sichuan, and partly overlaps with *P. leptosiphon* in southwestern Sichuan. Geographic separation may have driven *P. siphonantha* to diverge from the remaining species in this clade. Moreover, the carmine speckles on the lower lobes of *P. tenuituba* may appear distinct from *P. leptosiphon* to bumblebee pollinators where they both occur. It is also worth noting that *P. tenuituba* mainly grows in humid grasslands, but *P. leptosiphon* prefers to grow in the sandy, dry meadows. Niche specification and difference in pollinators may mediate reproductive isolation between *P. tenuituba* and *P. leptosiphon*. However, occasional hybridization might have been responsible for producing the suspected hybrid *P. dolichosiphon*.

The mountainous terrain of the home range of the *P. siphonantha* complex likely maintains genetic isolation among geographically isolated species. For example, *P. dolichantha* and its sister species *P. sigmoidea* are distributed in isolated mountains near Huize and Eryuan, Yunnan, while species closely related to them, *P. humilis* and *P. variegata*, occur in the Gaoligong Mountains and southwest Yunnan and Muli, Sichuan, respectively. *P. milliana* is widely distributed in northwestern Yunnan, and its sister *Pedicularis* sp. 2 (Jiaozi Snow Mountain) is only found in the Jiaozi Snow Mountain, Dongchuan, Yunnan. Of the seven species in this clade, *P. milliana* and *Pedicularis* sp. 1 co-occur in the Yulong Snow Mountain and *P. sigmoidea* has been collected from the south margin of the distribution range of *P. milliana* in Heqing, Eryuan, and Dali. Therefore, geographical isolation likely drove species divergence in this complex, with the exception that introgression between *P. milliana* and *P. sigmoidea* may have produced the suspected hybrid *Pedicularis* sp. 1 and the plastome capture of *P. milliana* from *P. sigmoidea* in Lijiang and south Shangri-La, northwest Yunnan.

## Conclusion

Overall, phylogenetic analyses of five DNA loci (nrITS, *matK*, *rbcL*, *trnH-psbA*, and *trnL-F*) clarify species delimitation within the *P. siphonantha* complex. Differences in geographical distribution and altitude can be important supplementary indicators to identify species of the *P. siphonantha* complex despite the lack of diagnostic morphological characters in herbarium specimens. The *P. siphonantha* complex likely originated from allopatric speciation. The origin of *P. milliana* and *Pedicularis* sp. 1 in the Lijiang region was plausibly due to an ancestral hybridization event. The morphological, molecular, and biogeographic evidence support taxonomic recognition of *Pedicularis* sp. 2. To better understand the evolution of the *P. siphonantha* complex, further studies of phenotype and environmental factors are needed.

## Data Availability Statement

The datasets presented in this study can be found in online repositories. The names of the repository/repositories and accession number(s) can be found in the article/Supplementary Material.

## Author Contributions

W-BY, HW, and D-ZL conceived the study. W-BY, HW, J-BY, and RC collected the data. RL and W-BY analyzed the data. RL, W-BY, CR, and D-ZL interpreted the results. All authors wrote and revised the article and approved the final version of the manuscript.

## Conflict of Interest

The authors declare that the research was conducted in the absence of any commercial or financial relationships that could be construed as a potential conflict of interest. The reviewer C-LX declared a shared affiliation, with no collaboration, with several of the authors HW, J-BY, and D-ZL to the handling editor at the time of the review.

## Publisher’s Note

All claims expressed in this article are solely those of the authors and do not necessarily represent those of their affiliated organizations, or those of the publisher, the editors and the reviewers. Any product that may be evaluated in this article, or claim that may be made by its manufacturer, is not guaranteed or endorsed by the publisher.

## Supplementary Material

Supplementary Figure 1 Maximum clade credibility tree of *Pedicularis* from BEAST divergence time analysis. The secondary calibration of the Orobanchaceae crown was constrained to 56 ± 10 Mya which was obtained from http://timetree.org/. The estimated age of nodes is presented above the branch. Node bars represent the 95% highest posterior density (HPD) interval.

## References

[B1] Acosta M. C. Premoli A. C. (2010). Evidence of chloroplast capture in South American *Nothofagus* (subgenus *Nothofagus*, Nothofagaceae). *Mol. Phylogenet. Evol.* 54 235–242. 10.1016/j.ympev.2009.08.008 19683588

[B2] Alfaro M. E. Zoller S. Lutzoni F. (2003). Bayes or bootstrap? a simulation study comparing the performance of bayesian Markov chain Monte Carlo sampling and bootstrapping in assessing phylogenetic confidence. *Mol. Biol. Evol.* 20 255–266. 10.1093/molbev/msg028 12598693

[B3] Bickford D. Lohman D. J. Sodhi N. S. Ng P. K. Meier R. Winker K. (2007). Cryptic species as a window on diversity and conservation. *Trends Ecol. Evol.* 22 148–155. 10.1016/j.tree.2006.11.004 17129636

[B4] Bouckaert R. R. Drummond A. J. (2017). bModelTest: bayesian phylogenetic site model averaging and model comparison. *BMC Evol. Biol.* 17:42. 10.1186/s12862-017-0890-6 28166715 PMC5294809

[B5] Bouckaert R. Heled J. Kuehnert D. Vaughan T. Wu C.-H. Xie D. (2014). BEAST 2: a software platform for bayesian evolutionary analysis. *PLoS Comp. Biol.* 10:e1003537. 10.1371/journal.pcbi.1003537 24722319 PMC3985171

[B6] Boufford D. E. (2014). Biodiversity hotspot: china’s hengduan mountains. *Arnoldia (Jamaica Plain)* 72 24–35.

[B7] Buckley T. R. Cordeiro M. Marshall D. C. Simon C. (2006). Differentiating between hypotheses of lineage sorting and introgression in New Zealand alpine cicadas (Maoricicada Dugdale). *Syst. Biol.* 55 411–425. 10.1080/10635150600697283 16684720

[B8] Darriba D. Posada D. Kozlov A. M. Stamatakis A. Morel B. Flouri T. (2020). ModelTest-NG: a new and scalable tool for the selection of DNA and protein evolutionary models. *Mol. Biol. Evol.* 37 291–294. 10.1093/molbev/msz189 31432070 PMC6984357

[B9] Degnan J. H. Rosenberg N. A. (2009). Gene tree discordance, phylogenetic inference and the multispecies coalescent. *Trends Ecol. Evol.* 24 332–340. 10.1016/j.tree.2009.01.009 19307040

[B10] Ding W.-N. Ree R. H. Spicer R. A. Xing Y.-W. (2020). Ancient orogenic and monsoon-driven assembly of the world’s richest temperate alpine flora. *Science* 369 578–581. 10.1126/science.abb4484 32732426

[B11] Don D. Hamilton F. Wallich N. (1825). *Prodromus Florae Nepalensis: Sive Enumeratio Vegetabilium Quae In Itinere Per Nepaliam Proprie Dictam Et Regiones Conterminas, Ann. 1802-1803.* Londini: J. Gale.

[B12] Eaton D. A. R. Fenster C. B. Hereford J. Huang S.-Q. Ree R. H. (2012). Floral diversity and community structure in *Pedicularis* (Orobanchaceae). *Ecology* 93 S182–S194. 10.1890/11-0501.1

[B13] Farnsworth A. Lunt D. J. Robinson S. A. Valdes P. J. Roberts W. H. G. Clift P. D. (2019). Past East Asian monsoon evolution controlled by paleogeography, not CO2. *Sci. Adv.* 5:eaax1697. 10.1126/sciadv.aax1697 31692956 PMC6821471

[B14] Favre A. Michalak I. Chen C. H. Wang J. C. Pringle J. S. Matuszak S. (2016). Out-of-Tibet: the spatio-temporal evolution of Gentiana (*Gentianaceae*). *J. Biogeogr.* 43 1967–1978. 10.1111/jbi.12840

[B15] Favre A. Paeckert M. Pauls S. U. Jaehnig S. C. Uhl D. Michalak I. (2015). The role of the uplift of the qinghai-tibetan plateau for the evolution of *Tibetan biotas*. *Biol. Rev.* 90 236–253. 10.1111/brv.12107 24784793

[B16] Hebert P. D. N. Gregory T. R. (2005). The promise of DNA barcoding for taxonomy. *Syst. Biol.* 54 852–859. 10.1080/10635150500354886 16243770

[B17] Hebert P. D. N. Cywinska A. Ball S. L. Dewaard J. R. (2003). Biological identifications through DNA barcodes. *Proc. Roy. Soc. Lond. B. Biol.* 270 313–321. 10.1098/rspb.2002.2218 12614582 PMC1691236

[B18] Hillis D. M. Bull J. J. (1993). An empirical test of bootstrapping as a method for assessing confidence in phylogenetic analysis. *Syst. Biol.* 42 182–192. 10.1093/sysbio/42.2.182

[B19] Hollingsworth P. M. Li D. Z. Van Der Bank M. Twyford A. D. (2016). Telling plant species apart with DNA: from barcodes to genomes. *Philos. Trans. R. Soc. Lond. B. Biol. Sci.* 371:20150338. 10.1098/rstb.2015.0338 27481790 PMC4971190

[B20] Hong W. Li D. Z. (2005). Pollination biology of four *Pedicularis* species (*Scrophulariaceae*) in northwestern Yunnan, China. *Ann. Mo. Bot. Gard.* 92 127–138.

[B21] Hoorn C. Mosbrugger V. Mulch A. Antonelli A. (2013). Biodiversity from mountain building. *Nat. Geosci.* 6 154–154. 10.1038/ngeo1742

[B22] Huang S.-Q. Fenster C. B. (2007). Absence of long-proboscid pollinators for long-corolla-tubed Himalayan *Pedicularis* species: implications for the evolution of corolla length. *Int. J. Plant Sci.* 168 325–331. 10.1086/510209

[B23] Huang S.-Q. Wang X.-P. Sun S.-G. (2016). Are long corolla tubes in Pedicularis driven by pollinator selection? *J. Integr. Plant Biol.* 58 698–700. 10.1111/jipb.12460 26714618

[B24] Huson D. H. Bryant D. (2006). Application of phylogenetic networks in evolutionary studies. *Mol. Biol. Evol.* 23 254–267. 10.1093/molbev/msj030 16221896

[B25] IBM (2017). *IBM SPSS Statistics for Windows. Version 25.0.* Armonk, NY: IBM Corp.

[B26] Joly S. Mclenachan P. A. Lockhart P. J. (2009). A statistical approach for distinguishing hybridization and incomplete lineage sorting. *Am. Nat.* 174 E54–E70. 10.1086/600082 19519219

[B27] Katoh K. Rozewicki J. Yamada K. D. (2019). MAFFT online service: multiple sequence alignment, interactive sequence choice and visualization. *Briefings Bioinf.* 20 1160–1166. 10.1093/bib/bbx108 28968734 PMC6781576

[B28] Kearse M. Moir R. Wilson A. Stones-Havas S. Cheung M. Sturrock S. (2012). Geneious Basic: an integrated and extendable desktop software platform for the organization and analysis of sequence data. *Bioinformatics* 28 1647–1649. 10.1093/bioinformatics/bts199 22543367 PMC3371832

[B29] Kolaczkowski B. Thornton J. W. (2007). Effects of branch length uncertainty on bayesian posterior probabilities for phylogenetic hypotheses. *Mol. Biol. Evol.* 24 2108–2118. 10.1093/molbev/msm141 17636043

[B30] Kress W. J. (2017). Plant DNA barcodes: applications today and in the future. *J. Syst. Evol.* 55 291–307. 10.1111/jse.12254

[B31] Kumar S. Stecher G. Li M. Knyaz C. Tamura K. (2018). MEGA X: molecular evolutionary genetics analysis across computing platforms. *Mol. Biol. Evol.* 35 1547–1549. 10.1093/molbev/msy096 29722887 PMC5967553

[B32] Lai Q. Ding L. Wang H. Yue Y. Cai F. (2007). Constraining the stepwise migration of the eastern Tibetan Plateau margin by apatite fission track thermochronology. *Sci. China Earth Sci.* 50 172–183. 10.1007/s11430-007-2048-7

[B33] Lam-Tung N. Schmidt H. A. Von Haeseler A. Bui Quang M. (2015). IQ-TREE: a fast and effective stochastic algorithm for estimating maximum-likelihood phylogenies. *Mol. Biol. Evol.* 32 268–274. 10.1093/molbev/msu300 25371430 PMC4271533

[B34] Lemey P. Rambaut A. Drummond A. J. Suchard M. A. (2009). Bayesian phylogeography finds its roots. *PLoS Comp. Biol.* 5:e1000520. 10.1371/journal.pcbi.1000520 19779555 PMC2740835

[B35] Li H.-L. (1948). A revision of the genus Pedicularis in China. part I. *Proc. Acad. Nat. Sci. Phila.* 100 205–378.

[B36] Li H.-L. (1949). A revision of the genus Pedicularis in China. part II. *Proc. Acad. Nat. Sci. Phila.* 101 1–378.

[B37] Liu B.-B. Campbell C. S. Hong D.-Y. Wen J. (2020). Phylogenetic relationships and chloroplast capture in the Amelanchier-Malacomeles-Peraphyllum clade (Maleae, Rosaceae): Evidence from chloroplast genome and nuclear ribosomal DNA data using genome skimming. *Mol. Phylogenet. Evol.* 147:106784. 10.1016/j.ympev.2020.106784 32135308

[B38] Liu M.-L. Yu W.-B. Wang H. (2013). Rapid identification of plant species and iFlora application of DNA barcoding in a large temperate genus *Pedicularis* (Orobanchaceae). *Plant Divers.* 35 707–714. 10.7677/ynzwyj201313168

[B39] Liu X. Wang Z. Shao W. Ye Z. Zhang J. (2017). Phylogenetic and taxonomic status analyses of the Abaso section from multiple nuclear genes and plastid fragments reveal new insights into the North America origin of *Populus* (*Salicaceae*). *Front. Plant Sci.* 7:2022. 10.3389/fpls.2016.02022 28101098 PMC5209371

[B40] Liu Y. Ye J.-F. Hu H.-H. Peng D.-X. Zhao L.-N. Lu L.-M. (2021). Influence of elevation on bioregionalisation: a case study of the Sino-Himalayan flora. *J. Biogeogr.* 48 2578–2587. 10.1111/jbi.14222

[B41] Macior L. W. (1990). Pollination ecology of Pedicularis punctata Decne. (*Scrophulariaceae*) in the Kashmir Himalaya. *Plant Species Biol.* 5 215–223. 10.1111/j.1442-1984.1990.tb00181.x

[B42] Macior L. W. Tang Y. (1997). A preliminary study of the pollination ecology of *Pedicularis* in the Chinese Himalaya. *Plant Species Biol.* 12 1–7. 10.1111/j.1442-1984.1997.tb00150.x

[B43] Macior L. W. Tang Y. Zhang J.-C. (2001). Reproductive biology of *Pedicularis* (*Scrophulariaceae*) in the Sichuan Himalaya. *Plant Species Biol.* 16 83–89. 10.1046/j.1442-1984.2001.00048.x

[B44] Maddison W. P. Maddison D. R. (2019). *Mesquite: A Modular System For Evolutionary Analysis. V3.7.0 [Online].* Available online at: http://www.mesquiteproject.org/ [Accessed August 10, 2020].

[B45] Maximowicz C. J. (1888). Diagnoses plantarum novarum Asiaticarum. *Bull. Acad. Sci. St Petersburg* 32 427–629.

[B46] Rambaut A. Drummond A. J. Xie D. Baele G. Suchard M. A. (2018). Posterior summarization in bayesian phylogenetics using Tracer 1.7. *Syst. Biol.* 67 901–904. 10.1093/sysbio/syy032 29718447 PMC6101584

[B47] Ree R. H. (2005). Phylogeny and the evolution of floral diversity in *Pedicularis* (*Orobanchaceae*). *Int. J. Plant Sci.* 166 595–613. 10.1086/430191

[B48] Rieseberg L. H. Whitton J. Linder C. R. (1996). Molecular marker incongruence in plant hybrid zones and phylogenetic trees. *Acta Bot. Neerl.* 45 243–262. 10.1111/j.1438-8677.1996.tb00515.x

[B49] Rokas A. Williams B. L. King N. Carroll S. B. (2003). Genome-scale approaches to resolving incongruence in molecular phylogenies. *Nature* 425 798–804. 10.1038/nature02053 14574403

[B50] Ronquist F. Teslenko M. Van Der Mark P. Ayres D. L. Darling A. Hohna S. (2012). MrBayes 3.2: efficient bayesian phylogenetic inference and model choice across a large model space. *Syst. Biol.* 61 539–542. 10.1093/sysbio/sys029 22357727 PMC3329765

[B51] Shimodaira H. (2002). An approximately unbiased test of phylogenetic tree selection. *Syst. Biol.* 51 492–508. 12079646 10.1080/10635150290069913

[B52] Shimodaira H. Hasegawa M. (1999). Multiple comparisons of log-likelihoods with applications to phylogenetic inference. *Mol. Biol. Evol.* 16 1114–1116. 10.1093/oxfordjournals.molbev.a026201

[B53] Stamatakis A. (2014). RAxML version 8: a tool for phylogenetic analysis and post-analysis of large phylogenies. *Bioinformatics* 30 1312–1313. 10.1093/bioinformatics/btu033 24451623 PMC3998144

[B54] Stegemann S. Keuthe M. Greiner S. Bock R. (2012). Horizontal transfer of chloroplast genomes between plant species. *Proc. Natl. Acad. Sci. U.S.A.* 109 2434–2438. 10.1073/pnas.1114076109 22308367 PMC3289295

[B55] Struck T. H. Feder J. L. Bendiksby M. Birkeland S. Cerca J. Gusarov V. I. (2018). Finding evolutionary processes hidden in cryptic species. *Trends Ecol. Evol.* 33 153–163. 10.1016/j.tree.2017.11.007 29241941

[B56] Stull G. W. Soltis P. S. Soltis D. E. Gitzendanner M. A. Smith S. A. (2020). Nuclear phylogenomic analyses of asterids conflict with plastome trees and support novel relationships among major lineages. *Am. J. Bot.* 107 790–805. 10.1002/ajb2.1468 32406108

[B57] Tsoong P.-C. (1955). A new system for the genus Pedicularis. *Acta Phytotax. Sin.* 4 71–147.

[B58] Tsoong P.-C. (1956). A new system for the genus Pedicularis. *Acta Phytotax. Sin.* 5 41–73 239–278.

[B59] Tsoong P.-C. (1963). “Scrophulariaceae (Pars II),” in *Flora Reipublicae Popularis Sinacae*, Vol. 68 eds Chien S.-S. Chun W.-Y. (Beijing: Science Press), 61–378.

[B60] Wang E. Kirby E. Furlong K. P. Van Soest M. Xu G. Shi X. (2012). Two-phase growth of high topography in eastern Tibet during the Cenozoic. *Nat. Geosci.* 5 640–645. 10.1038/ngeo1538

[B61] Xing Y. Ree R. H. (2017). Uplift-driven diversification in the Hengduan Mountains, a temperate biodiversity hotspot. *Proc. Natl. Acad. Sci. U.S.A.* 114 E3444–E3451. 10.1073/pnas.1616063114 28373546 PMC5410793

[B62] Yang C.-F. Gituru R. W. Guo Y.-H. (2007). Reproductive isolation of two sympatric louseworts, *Pedicularis rhinanthoides* and *Pedicularis longiflora* (Orobanchaceae): how does the same pollinator type avoid interspecific pollen transfer? *Biol. J. Linn. Soc.* 90 37–48. 10.1111/j.1095-8312.2007.00709.x

[B63] Yang F. S. Qin A. L. Li Y. F. Wang X. Q. (2012). Great genetic differentiation among populations of *Meconopsis integrifolia* and its implication for plant speciation in the Qinghai-Tibetan Plateau. *PLoS One* 7:e37196. 10.1371/journal.pone.0037196 22590654 PMC3349641

[B64] Yang H.-B. Holmgren N. H. Mill R. R. (1998). “Pedicularis Linn,” in *Flora of China*, Vol. 18 eds Wu Z.-Y. Raven P. H. (St. Louis, MI: Missouri Botanical Garden Press & Science Press), 97–209. 10.1016/j.biopha.2017.10.133

[B65] Ye X. Y. Ma P. F. Guo C. Li D. Z. (2021). Phylogenomics of *Fargesia* and *Yushania* reveals a history of reticulate evolution. *J. Syst. Evol.* 59 1183–1197. 10.1111/jse.12719

[B66] Yi T.-S. Jin G.-H. Wen J. (2015). Chloroplast capture and intra- and inter-continental biogeographic diversification in the Asian – New World disjunct plant genus Osmorhiza (*Apiaceae*). *Mol. Phylogenet. Evol.* 85 10–21. 10.1016/j.ympev.2014.09.028 25585153

[B67] Yu W. B. Huang P. H. Li D. Z. Wang H. (2013). Incongruence between nuclear and chloroplast DNA phylogenies in *Pedicularis* section *Cyathophora* (*Orobanchaceae*). *PLoS One* 8:e74828. 10.1371/journal.pone.0074828 24069353 PMC3777957

[B68] Yu W. B. Huang P.-H. Ree R. H. Liu M.-L. Li D.-Z. Wang H. (2011). DNA barcoding of *Pedicularis* L. (*Orobanchaceae*): evaluating four universal barcode loci in a large and hemiparasitic genus. *J. Syst. Evol.* 49 425–437. 10.1111/j.1759-6831.2011.00154.x

[B69] Yu W. B. Liu M. L. Wang H. Mill R. R. Ree R. H. Yang J. B. (2015). Towards a comprehensive phylogeny of the large temperate genus *Pedicularis* (*Orobanchaceae*), with an emphasis on species from the Himalaya-Hengduan Mountains. *BMC Plant Biol.* 15:176. 10.1186/s12870-015-0547-9 26159907 PMC4498522

[B70] Yu W. B. Wang H. Liu M. L. Grabovskaya-Borodina A. E. Li D. Z. (2018). Phylogenetic approaches resolve taxonomical confusion in *Pedicularis* (*Orobanchaceae*): reinstatement of *Pedicularis delavayi* and discovering a new species *Pedicularis milliana*. *PLoS One* 13:e0200372. 10.1371/journal.pone.0200372 30044806 PMC6059426

[B71] Zha H.-G. Milne R. I. Sun H. (2010). Asymmetric hybridization in Rhododendron agastum: a hybrid taxon comprising mainly F(1)s in Yunnan, China. *Ann. Bot.* 105 89–100. 10.1093/aob/mcp267 19887474 PMC2794068

[B72] Zhang D.-C. Ye J.-X. Sun H. (2016). Quantitative approaches to identify floristic units and centres of species endemism in the Qinghai-Tibetan Plateau, south-western China. *J. Biogeogr.* 43 2465–2476. 10.1111/jbi.12819

[B73] Zhang Y.-Z. Replumaz A. Leloup P. H. Wang G.-C. Bernet M. Van Der Beek P. (2017). Cooling history of the Gongga batholith: implications for the Xianshuihe Fault and Miocene kinematics of SE Tibet. *Earth Planet. Sci. Lett.* 465 1–15. 10.1016/j.epsl.2017.02.025

