# Supplementary figures and images for: Cryptic Species Diversification of the Pedicularis siphonantha Complex (Orobanchaceae) in the Mountains of Southwest China Since the Pliocene
Source: Front Plant Sci. 2022 Mar 24;13:811206. doi: 10.3389/fpls.2022.811206 (PMC8987768; doi:10.3389/fpls.2022.811206)

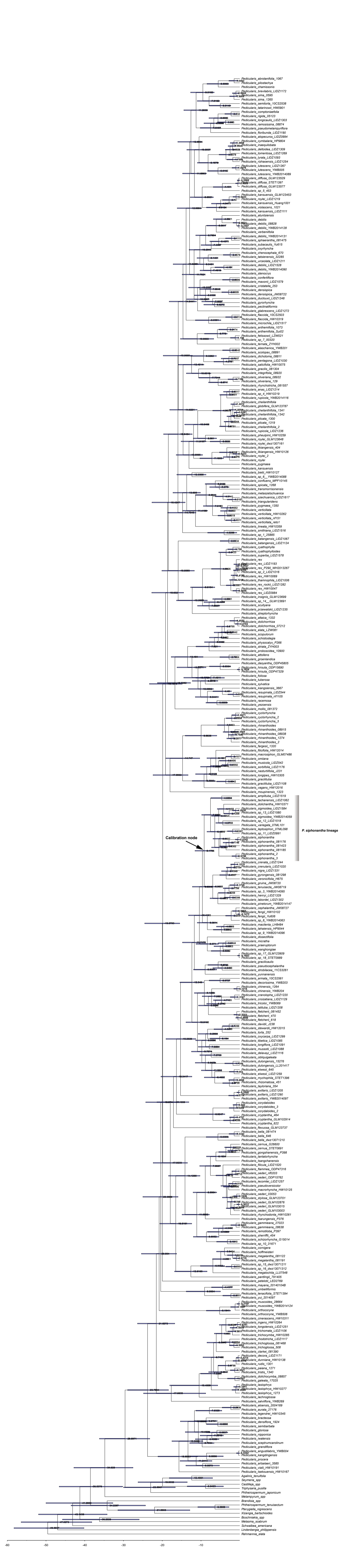

Supplement: Supplementary Figure 1 — Maximum clade credibility tree of Pedicularis from BEAST divergence time analysis. The secondary calibration of the Orobanchaceae crown was constrained to 56 ± 10 Mya which was obtained from http://timetree.org/. The estimated age of nodes is presented above the branch. Node bars represent the 95% highest posterior density (HPD) interval. [file Data_Sheet_1.PDF]
